# Optimal input design for multibody systems by using an extended adjoint approach

**DOI:** 10.1007/s11044-016-9541-8

**Published:** 2016-10-05

**Authors:** Stefan Oberpeilsteiner, Thomas Lauss, Karin Nachbagauer, Wolfgang Steiner

**Affiliations:** 1 Faculty of Engineering and Environmental Sciences, University of Applied Sciences Upper Austria, Stelzhamerstrasse 23, 4600 Wels, Austria; 2 Institute of Mechanics and Mechatronics, Vienna University of Technology, Getreidemarkt 9/325, 1060 Wien, Austria

**Keywords:** Optimal input design, Parameter identification, Adjoint method, Design of experiment, Sensitivity analysis

## Abstract

We present a method for optimizing inputs of multibody systems for a subsequently performed parameter identification. Herein, optimality with respect to identifiability is attained by maximizing the information content in measurements described by the Fisher information matrix. For solving the resulting optimization problem, the adjoint system of the sensitivity differential equation system is employed. The proposed approach combines these two well-established methods and can be applied to multibody systems in a systematic, automated manner. Furthermore, additional optimization goals can be added and used to find inputs satisfying, for example, end conditions or state constraints.

## Introduction

The problem of optimal input design plays a key role when considering an experiment in order to perform a parameter identification. Poorly planned experiments can cause a waste of time and resources and yield little useful information. The linkage between the experiment and modeling world is called *design of experiment* (DOE). If the model knowledge is used for designing the experiments, then often the term *model-based DOE* can be found in the literature [5]. One of the first authors dealing with the topic of designing experiments was R.A. Fisher in his substantial work *The Design of Experiments* [4]. Although the importance and applicability onto the problem of optimal input design was known only to some extent, many recent papers refer to his work. Fisher stated that the basic problem of DOE is to decide which pattern of factor combination will best reveal the properties of the response and how this response is influenced by these factors. The term *optimal input design* emerges from the work of Mehra [10, 11], who worked on linear discrete-time systems. There, the most important requirement for designing an input was to generate a system output allowing one to determine system parameters featuring a minimum of variance. Morelli [12] developed a method for generating optimal input signals utilizing basic statistics, including the theory of maximum likelihood estimates for parameters. On this basis, Morelli [13] showed the practicability of the method, where system inputs for flight tests with an F-18 HARV (high alpha research vehicle) are determined. Recent publications include different fields of application, for example, Jauberthie et al. [7, 8] considered a model of an aerodynamic problem. Therein the ideas of Morelli [12] are used to generate an optimal input for identifying aerodynamic parameters of an aircraft. An extensive work on optimal input design in the field of chemistry and also on DOE in general was done by Franceschini [5]. According to this work, a *model-based DOE* is characterized by:

- The explicit use of the model equations (including any constraint) and current parameters to predict the “information content” of the next experiment (through the evaluation of some suitable objective function), and
- the application of an optimization framework to find a numerical solution of the resulting problem.

Another application dealing with optimal input design is that of process control. Chianeh [3] investigated models of tank systems fed by a pump. The goal was to determine the flow exponent used in Bernoulli’s law. Keesman [9] considered optimal input design for choosing between model structures or *model discrimination*. A further topic related to optimal input design is that of optimal sensor placement. In the work by Castro-Triguero [2], several methodologies for computing a minimal set of sensor locations were investigated in order to get the required information for health monitoring of bridge structures.

The latter mentioned approaches cannot be applied directly to the model of mechanical systems. Therefore, in this paper, we show how the process of optimal input design can be systematically applied for mechanical systems. As the adjoint method provides outstanding performance in the field of optimal control, this method is used for computing the update direction during the optimal input iteration process. First, a proper performance measure or cost functional for determining optimal inputs is defined via statistical context. For further analysis, the system of sensitivity differential equations is derived, and also the adjoint system of the original system is extended by these new terms.

## Theoretical background

For simplicity, the model equations investigated in this work are first-order ordinary differential equations (ODE), and therefore a set of minimal coordinates is used. Nevertheless, it is possible to formulate the entire process for more general differential algebraic equations and therefore for models with redundant coordinates. The model equations can be written as

1 $$ \begin{aligned} \dot{\mathbf {x}} &= \mathbf {f}(\mathbf {x}, \mathbf {u}, \mathbf {b}, t), \quad \mathbf {x}(0) = \mathbf {x}_{0}, \\ \mathbf {y}&= \mathbf {y}(\mathbf {x}), \end{aligned} $$

where $\mathbf {x}(t) \in \mathbb{R}^{N_{x}}$ is the vector of state variables, $\mathbf {u}(t)\in \mathbb{R}^{N_{u}}$ is the vector of model inputs, and $\mathbf {b}\in \mathbb{R}^{n}$ is the vector of model parameters. In order to compare the simulation result with measurements, a vector of model outputs $\mathbf {y}(\mathbf {x})$ is defined such that it matches with measured outputs $\tilde {\mathbf {y}}(t) \in \mathbb{R}^{m}$.

### System sensitivity analysis

Analyzing a system reaction to small changes in the system parameters at a special time point $t_{i}$ results in the sensitivity matrix

2 $$ \mathbf {S}(t_{i}) = \begin{bmatrix} \mathbf {y}_{\mathbf {b}_{1}} & \mathbf {y}_{\mathbf {b}_{2}} & \ldots & \mathbf {y}_{\mathbf {b}_{n}} \end{bmatrix} . $$

In case of investigating a multibody system, this analysis can be performed by forming the derivatives of the output vector $\mathbf {y}$ with respect to the system parameters $\mathbf {b}$. Therefore, Eq. (1) is differentiated with respect to each parameter. In the following, the abbreviations $\mathbf {x}_{\mathbf {b}_{j}}$, $\mathbf {y}_{\mathbf {x}}$, $\mathbf {f}_{\mathbf {b}_{j}}$, and $\mathbf {f}_{\mathbf {x}}$ are used instead of $\frac{\partial \mathbf {x}}{\partial \mathbf {b}_{j}}$, $\frac{\partial \mathbf {y}}{ \partial \mathbf {x}}$, $\frac{\partial \mathbf {f}}{\partial \mathbf {b}_{j}}$, and $\frac{\partial \mathbf {f}}{\partial \mathbf {x}}$, respectively. Hence, the sensitivity equations read

3 $$ \begin{aligned} \dot{\mathbf {x}}_{\mathbf {b}_{j}} &= \mathbf {f}_{\mathbf {x}}\mathbf {x}_{\mathbf {b}_{j}} + \mathbf {f}_{\mathbf {b}_{j}}, \\ \mathbf {y}_{\mathbf {b}_{j}} &= \mathbf {y}_{\mathbf {x}} \mathbf {x}_{\mathbf {b}_{j}}, \end{aligned} $$

where $\mathbf {y}_{\mathbf {b}_{j}}(t_{i})$ equals the $j$th column in the output sensitivity matrix $\mathbf {S}(t_{i})$. In the case of more than one unknown parameter, the system of differential equations for the state variables and for the sensitivities can be written by

4 $$\begin{aligned} \begin{bmatrix} \dot{\mathbf {x}} \\ \dot{\mathbf {x}}_{\mathbf {b}_{1}} \\ \dot{\mathbf {x}}_{\mathbf {b}_{2}} \\ \vdots \\ \dot{\mathbf {x}}_{\mathbf {b}_{n}} \end{bmatrix} = \dot{\mathbf {z}} = \begin{bmatrix} \mathbf {f}\\ \mathbf {f}_{\mathbf {x}}\mathbf {x}_{\mathbf {b}_{1}} + \mathbf {f}_{\mathbf {b}_{1}} \\ \mathbf {f}_{\mathbf {x}}\mathbf {x}_{\mathbf {b}_{2}} + \mathbf {f}_{\mathbf {b}_{2}} \\ \vdots \\ \mathbf {f}_{\mathbf {x}}\mathbf {x}_{\mathbf {b}_{n}} + \mathbf {f}_{\mathbf {b}_{n}} \end{bmatrix} = \tilde{\mathbf {f}}. \end{aligned}$$

The Jacobian $\tilde{\mathbf {f}}_{\mathbf {z}}$ of the extended system, which is required for further computations, reads

5 $$\begin{aligned} \tilde{\mathbf {f}}_{\mathbf {z}} = \begin{bmatrix} \mathbf {f}_{\mathbf {x}} & \mathbf {0}& \mathbf {0}& \ldots & \mathbf {0}\\ (\mathbf {f}_{\mathbf {x}}\mathbf {x}_{\mathbf {b}_{1}} + \mathbf {f}_{\mathbf {b}_{1}})_{\mathbf {x}} & \mathbf {f}_{\mathbf {x}} & \mathbf {0}& \ldots & \mathbf {0}\\ (\mathbf {f}_{\mathbf {x}}\mathbf {x}_{\mathbf {b}_{2}} + \mathbf {f}_{\mathbf {b}_{2}})_{\mathbf {x}} & \mathbf {0}& \mathbf {f}_{\mathbf {x}} & \ldots & \mathbf {0}\\ \vdots \\ (\mathbf {f}_{\mathbf {x}}\mathbf {x}_{\mathbf {b}_{n}} + \mathbf {f}_{\mathbf {b}_{n}})_{\mathbf {x}} & \mathbf {0}& \mathbf {0}& \ldots & \mathbf {f}_{\mathbf {x}} \end{bmatrix} . \end{aligned}$$

### Maximization of the information content in experimental measurement data

When dealing with optimal input design, first of all, the term “optimality” has to be clarified. As Morelli defined in [12], optimal inputs minimize the parameter standard errors during model parameter estimation with a maximum likelihood estimator. In other words, the information contained in experimental measurement data has to be maximized. Hence, a proper measure, or cost functional, can be constructed by using a norm of the Fisher information matrix ℳ (see [12]):

6 $$ \mathcal {M}= \sum_{i=1}^{N_{s}}\mathbf {S}(t_{i})^{T}\mathbf {R}^{-1}\mathbf {S}(t_{i}). $$

Here, $N_{s}$ is the number of samples taken during the measurement, and $\mathbf {R}$ is the discrete noise covariance matrix, which is unknown prior to the optimization process. A very common way is to assume no correlation among the system outputs and moreover that the variance of all system outputs is equal. Using this assumption, $\mathbf {R}$ reduces to the identity matrix $\mathbf {I}$.

In [1], several norms or optimality metrics are suggested for optimal input design. Investigating the determinant (*D-optimality*) or eigenvalues (*E-optimality*) of ℳ in a cost functional does not allow us to apply straightforward variational calculus. Hence, the so called *A-optimality* is chosen, which incorporates the trace of ℳ. Moreover, common optimization algorithms search for the minimum of a cost functional $J(\mathbf {u})$. Therefore, the maximization of the information content leads to a cost functional using the negative trace of ℳ.

For further derivations, the cost functional is defined as a continuous function. Instead of forming the sum in Eq. (6), the inner product of columns of $\mathbf {S}$ is integrated over time. The resulting cost functional to be minimized then reads

7 $$ J= - \int_{t_{0}}^{t_{f}}\sum_{j=1}^{n} \mathbf {y}_{\mathbf {b}_{j}}(t)^{\mathsf {T}}\mathbf {y}_{\mathbf {b}_{j}}(t) \mathrm {d}t. $$

### The adjoint method

Recalling that the cost functional in Eq. (7) uses the system sensitivities and hence outputs of the extended system of Eq. (4), optimal input design can be seen as the standard problem of optimal control for the extended system. In previous publications of the authors [14, 15], the adjoint method is presented to be the most efficient way to solve such problems. Since the cost functional in Eq. (7) depends on the system sensitivities and therefore on the states $\mathbf {z}$ of the extended system in Eq. (4), the cost functional can be defined as follows:

8 $$ J(\mathbf {u}) = \int_{t_{0}}^{t_{f}} h(\mathbf {z},\mathbf {u},t) \mathrm {d}t. $$

The problem is to find control variables $\mathbf {u}(t)$ that minimize this function. In order to provide a search direction for the optimization process, the variation of the cost functional with respect to the parameters has to be evaluated. The starting point of the adjoint method is to add the system equations in Eq. (4) to the integrand in Eq. (8). Hence, the extended cost functional reads

9 $$ J(\mathbf {u}) = \int_{t_{0}}^{t_{f}} \bigl[ h+\mathbf {p}^{\mathsf {T}}( \tilde{ \mathbf {f}}-\dot{\mathbf {z}}) \bigr] \mathrm {d}t. $$

Since the system equations are satisfied, the actual value of $J$ does not depend on the selection of the functions $\mathbf {p}(t)$. Introducing the Hamiltonian

10 $$ H(\mathbf {z},\mathbf {p},\mathbf {u},t) = h(\mathbf {z},\mathbf {u},t) + \mathbf {p}^{\mathsf {T}} \tilde{\mathbf {f}}(\mathbf {z},\mathbf {u},t), $$

Eq. (9) becomes

11 $$ J(\mathbf {u}) = \int_{t_{0}}^{t_{f}} \bigl[ H-\mathbf {p}^{\mathsf {T}}\dot{\mathbf {z}} \bigr] \mathrm {d}t. $$

For a given forward solution $\mathbf {z}(t)$ of the system equations (4) with control variables $\mathbf {u}(t)$ and fixed parameters $\mathbf {b}$, the variation of $\mathbf {u}$ about $\delta \mathbf {u}$ results in variations of $\mathbf {z}(t)$ about $\delta \mathbf {z}(t)$. This again results in a variation of the functional $J$ about $\delta J$. Considering the first-order terms only, $\delta J$ is given by

12 $$ \delta J= \int_{t_{0}}^{t_{f}} \bigl[ H_{\mathbf {u}}\delta \mathbf {u}+ H_{\mathbf {z}} \delta \mathbf {z}- \mathbf {p}^{\mathsf {T}} \delta \dot{\mathbf {z}} \bigr] \mathrm {d}t, $$

where $H_{\mathbf {u}}$ and $H_{\mathbf {z}}$ stand for the partial derivatives of $H$ with respect to the vector of system inputs $\mathbf {u}$ and states of the extended system $\mathbf {z}$, respectively. In order to avoid the computation of the variations of $\mathbf {z}$, the last term of Eq. (12) is integrated by parts, and therefore the variation of the cost functional reads

13 $$ \begin{aligned}[b] \delta J&= \int_{t_{0}}^{t_{f}} \bigl[ H_{\mathbf {u}}\delta \mathbf {u}+ H _{\mathbf {z}}\delta \mathbf {z}+ \dot{\mathbf {p}}^{\mathsf {T}}\delta \mathbf {z}\bigr] \mathrm {d}t- \mathbf {p}^{\mathsf {T}}\delta \mathbf {z}|_{t_{0}}^{t_{f}} \\ &= \int_{t_{0}}^{t_{f}} \bigl[ H_{\mathbf {u}}\delta \mathbf {u}+ \bigl( H_{\mathbf {z}}+\dot{\mathbf {p}}^{\mathsf {T}} \bigr) \delta \mathbf {z}\bigr] \mathrm {d}t- \mathbf {p}(t_{f}) \delta \mathbf {z}(t_{f}). \end{aligned} $$

Herein, the variation $\delta \mathbf {z}(0)=0$ is already neglected as the initial conditions are prescribed independently from the actual choice of parameters. Now, in order to eliminate the term multiplied with $\delta \mathbf {z}$, a system of adjoint equations for the adjoint variables $\mathbf {p}(t)$ can be formed. The adjoint system reads

14 $$ \dot{\mathbf {p}} = -H_{\mathbf {z}}^{\mathsf {T}}\quad \text{and} \quad \mathbf {p}(t_{f}) = \mathbf {p}_{f}. $$

This set of equations may be solved backwards in time since there is only an initial condition at time $t = t_{f}$. At this point, there is no constraint on the adjoint states at $t = t_{f}$, and therefore they can be chosen arbitrarily. An option presented in [14] is to add a further term to the cost function, which allows us to consider end conditions for the system states $\mathbf {z}$. This term is commonly denoted as a *scrap-function*. Using Eq. (14), the variation of $J$ is given by Eq. (13):

15 $$ \delta J= \int_{t_{0}}^{t_{f}} H_{\mathbf {u}}\delta \mathbf {u}\mathrm {d}t. $$

In order to achieve the largest possible decrease of $\delta J$, $\delta \mathbf {u}(t)$ is chosen in the direction of $H_{\mathbf {u}}^{\mathsf {T}}$. Due to nonlinearities in the cost functional, this direction is only valid near the current system input $\mathbf {u}(t)$. Therefore, the update has to be done incrementally by using

16 $$ \delta \mathbf {u}(t) = -\kappa H_{\mathbf {u}}^{\mathsf {T}} $$

with small numbers $\kappa $. Finding a value for $\kappa $ that minimizes $J$ may be done by applying an optimization scheme such as the classical linesearch algorithm.

### Considering model input constraints

**Fig. 1:**
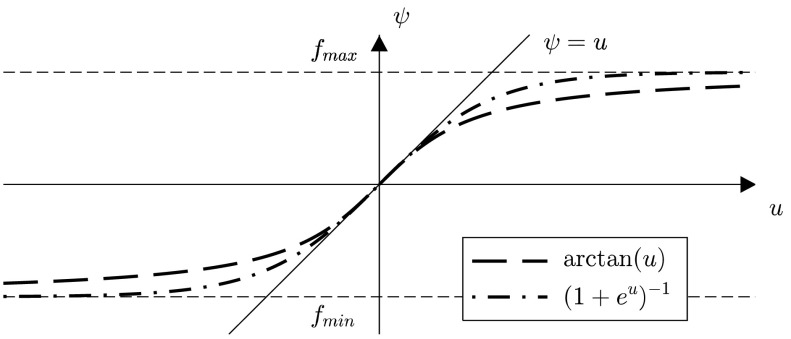
Comparison of constraining functions

In most cases, maximizing the information content in measurements leads to a maximization of the energy put into the system under consideration. Therefore, the system inputs to be optimized have to be constrained in a way that applicable optimization results are generated. One main difficulty is the direct influence of such constraints on the optimization process. They insert further nonlinearities and therefore affect the convergence negatively. The approach chosen in this work is to transform the input in such a way that the transition from unconstrained input $u_{i}$ to constrained input $\psi _{i}(u _{i})$ is very smooth. In order to satisfy $\psi _{i}(u_{i}) \in [u_{i}^{-},u_{i}^{+}]$, the authors in [6] propose to use the function

17 $$ \psi _{i}(u_{i}) = u_{i}^{+} - \frac{u_{i}^{+} - u_{i}^{-}}{1+ \exp (s u_{i})},\quad s=\frac{4}{u_{i}^{+} - u_{i}^{-}}, $$

for constraining the input $u_{i}$. The term $s$ is introduced in order to correct the slope at $u_{i} = 0$ to $\psi _{i}'(0) = 1$. Another possibility for constraining inputs is to use the arctangent function

18 $$ \psi _{i}(u_{i}) = \frac{u_{i}^{+} + u_{i}^{-}}{2} + s \arctan \biggl( \frac{1}{s} u_{i} \biggr) , \quad s = \frac{u_{i}^{+} - u _{i}^{-}}{\pi }, $$

again with $s$ being the function to correct the slope at $u_{i} = 0$. In Fig. 1, these two functions are displayed. As we prefer a function with distinct slopes in a wide range, the arctangent function is suggested as a constraining function, although the direct relation between $\psi _{i} \approx u _{i}$ is only valid in a very small region.

## Parameter identification

The purpose of optimal input design is to generate the excitation for a subsequent parameter estimation. Application of the computed optimal input onto the real system leads to an optimal desired trajectory. Therefore, in the following, an approach utilizing the system sensitivities derived in Sect. 2.1 is presented.

In direct comparison to the optimization of inputs in the previous section optimizing, the parameters is less expensive. First of all, a suitable function, measuring the error of the simulation with respect to the experiment, has to be specified. Choosing the root mean square (RMS) error

19 $$ E_{\text{RMS}}(\mathbf {b}) = \sum_{i=1}^{N_{s}} \frac{1}{2} \boldsymbol {\varDelta }\boldsymbol {y}_{i}^{\mathsf {T}}\boldsymbol {\varDelta }\boldsymbol {y}_{i},\quad \boldsymbol {\varDelta }\boldsymbol {y}_{i} = \begin{bmatrix} \tilde{\mathbf {y}}_{1,i} - \mathbf {y}_{1}(t_{i}) \\ \tilde{\mathbf {y}}_{2,i} - \mathbf {y}_{2}(t_{i}) \\ \vdots \\ \tilde{\mathbf {y}}_{m,i} - \mathbf {y}_{m}(t_{i}) \end{bmatrix} , $$

simplifies further derivations for the optimization procedure. Here, $N_{s}$ is the number of sampling points in the measurement, and $\boldsymbol {\varDelta }\boldsymbol {y}$ is the deviation from simulation data $\mathbf {y}$ to measured data $\tilde {\mathbf {y}}$ at a time point $t_{i}$. When arranging the output sensitivities at this time point in the form of the sensitivity matrix Eq. (2), the gradient of $E_{\text{RMS}}(\mathbf {b})$ can be written as

20 $$ \nabla E_{\text{RMS}}(\mathbf {b}) = -\sum_{i=1}^{N_{s}} \boldsymbol {\varDelta }\boldsymbol {y}_{i}^{\mathsf {T}} \mathbf {S}(t _{i}). $$

Performing another differentiation on $\nabla E_{\text{RMS}}$ and neglecting higher-order terms, the Hessian reads

21 $$ \nabla^{2}E_{\text{RMS}}= \sum_{i=1}^{N_{s}} \biggl( \mathbf {S}(t_{i})^{\mathsf {T}} \mathbf {S}(t _{i}) - \frac{\partial }{\partial \mathbf {b}} \bigl( \boldsymbol {\varDelta }\boldsymbol {y}^{\mathsf {T}}(t_{i}) \mathbf {S}(t _{i}) \bigr) \biggr) \approx \sum_{i=1}^{N_{s}} \mathbf {S}(t_{i})^{\mathsf {T}} \mathbf {S}(t_{i}), $$

in which $\boldsymbol {\varDelta }\boldsymbol {y}^{\mathsf {T}}(t_{i})$ is considered as constant in the case of the second term. With the exact solution for $\nabla E_{\text{RMS}}$ and an approximation for $\nabla^{2} E_{\text{RMS}}$, the Newton method may be applied. Performing a sufficient number of iterations

22 $$ \varDelta \mathbf {b}_{k+1} = -\bigl(\nabla^{2} E_{\text{RMS}}(\mathbf {b}_{k})\bigr)^{-1} \nabla E_{\text{RMS}}(\mathbf {b}_{k}), $$

the optimal set of parameters can be determined.

## Numerical examples

### Two-mass oscillator

**Fig. 2:**
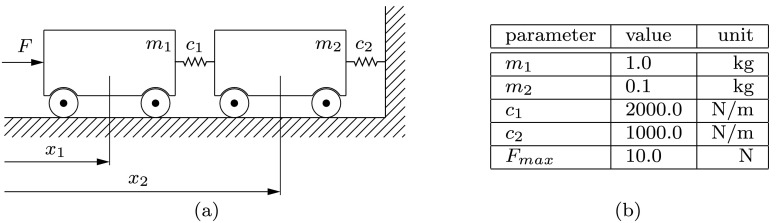
An excitation signal $F$ for the two-mass oscillator in (**a**) is searched. In (**b**), the parameters necessary for the numerical simulation are specified

As an introductory example, the relatively simple model of the two-mass oscillator in Fig. 2(a) is analyzed. The mass $m_{1}$ is excited by the force $F(t)$, whereas this force is constrained to a maximum amplitude of $F_{\mathrm{max}}$ by means of the constraining function presented in Sect. 2.4. Assuming that the position $x_{2}$ is measured during an experiment, a time history $F(t_{i})$ maximizing the information content with respect to the stiffness $c_{2}$ should be computed at discrete time points $t_{i} = \{t_{0}, t_{1}, \dots , t_{f}\}$, with $t_{f} = 1\mbox{ s}$. For starting the iterative optimization process, the initial input is set to a constant value $F_{0}(t_{i}) = 0.5 \cdot F_{\mathrm{max}}$. The parameter setting used can be found in Fig. 2(b). In order to avoid a motion of the bodies at $t > t_{f}$, a scrap function mentioned in Sect. 2.3 is specified such that the end velocities are set to $v_{1}(t_{f}) = v_{2}(t_{f}) = 0$.

**Fig. 3:**
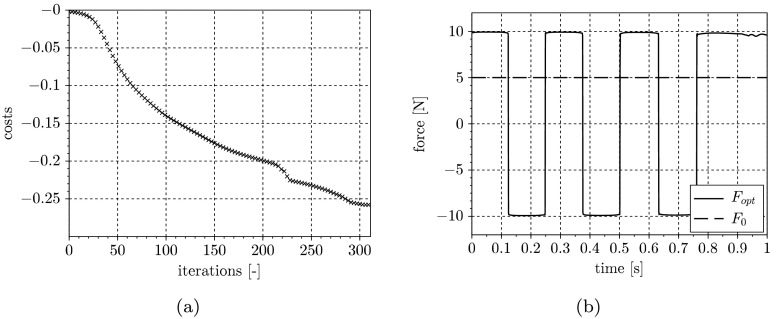
Convergence history for input optimization (**a**) and resulting excitation signal (**b**)

In Fig. 3(a), the convergence history for the input optimization, and in Fig. 3(b), the resulting constrained input signal is depicted. Due to the linear convergence rate of the gradient method, the convergence history shows quite poor but stable behavior.

**Fig. 4:**
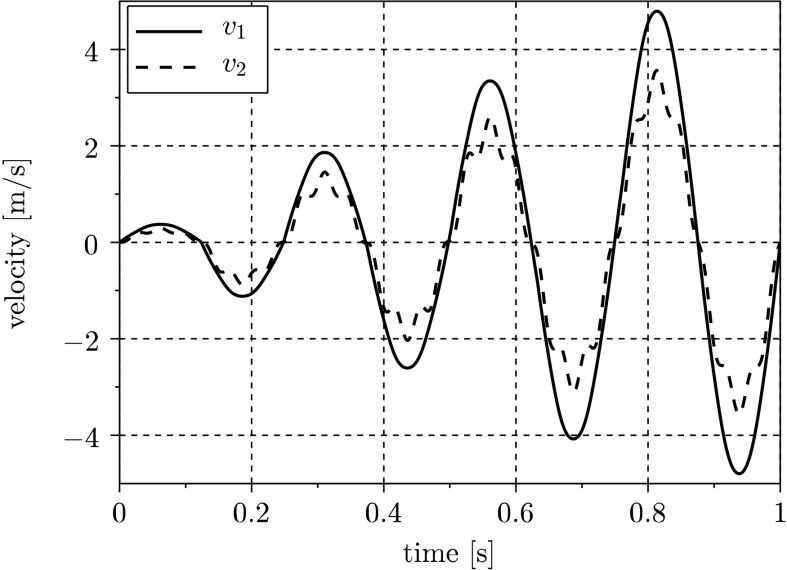
Velocity $v_{1}$ and $v_{2}$ for $F_{\mathrm{opt}}$ observing end conditions

**Fig. 5:**
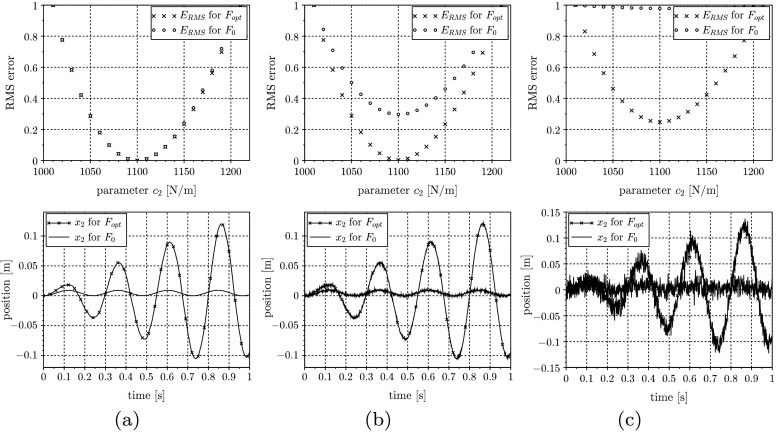
Evaluation of $E_{\text{RMS}}$ and $x_{2}(t)$ using $F_{0}$ and $F_{\mathrm{opt}}$ for different standard deviations $\sigma = 0$ (**a**), $\sigma = 10^{-6}$ (**b**), and $\sigma = 10^{-4}$ (**c**)

As this is only an example without a physical realization, the measurements necessary for the parameter identification are generated by simulation using the optimized excitation force $F_{\mathrm{opt}}$ and the assumed stiffness coefficient $\bar{c_{2}} = 1100\mbox{ N/m}$. Due to various mechanisms, real sensor recordings provide biased signals. Therefore, the generated “measurements” are superimposed with zero-mean Gaussian noise. In Fig. 5, these measurements are presented for different standard deviations. For comparison, also the measurement using the initial excitation signal $F_{0}$ is displayed, which features smaller amplitudes and obviously leads to uncertainties of the parameter identification process. The velocity plot in Fig. 4 shows that the desired end conditions for $v_{1}$ and $v_{2}$ are fulfilled in the case of using $F_{\mathrm{opt}}$.

Now, the main aim of optimal input design is to improve the quality of the parameter identification result, and as a side benefit, some constraints on the system states and inputs can be regarded. In order to show the advantage of the input generation for the actual example in Fig. 5, the RMS error evaluated for parameter values $c_{2}$ near $\bar{c_{2}}$ is shown. According to the left plot of RMS errors in Fig. 5(a), utilizing $F_{\mathrm{opt}}$ does not improve the shape of the cost functional in comparison to $F_{0}$ when using the unbiased measurements. The more noisy the sensor recording used for computing $E_{\text{RMS}}$, the more advantageous the optimal input $F_{\mathrm{opt}}$ influences the shape of the RMS error used for parameter identification. Since this is only a one-dimensional optimization problem, no real benefit with regard to a speed up of the optimization process can be gained. Advantages may be the increased curvature of $E_{\text{RMS}}$ and the possibility to include end conditions on the system outputs.

### Cart pendulum system

**Fig. 6:**
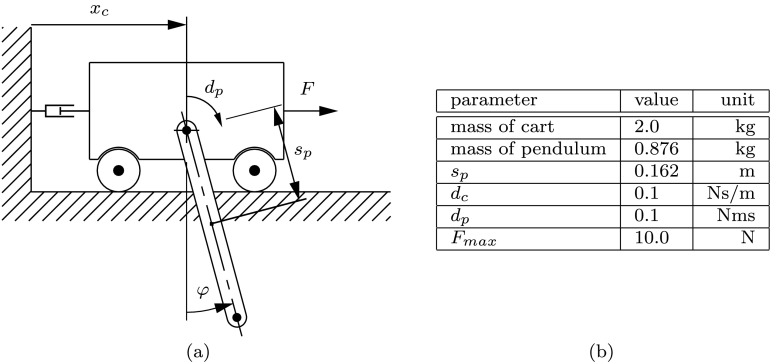
An optimal input $F$ is searched for the cart pendulum system in (**a**). In (**b**), the parameters necessary for the numerical simulation are specified

A system consisting of a translational moving cart and a pendulum mounted at its center of mass is studied next. Figure 6(a) shows the geometric description of the cart pendulum system. The cart is only allowed to move along the $x$-axis leading to a two-dimensional motion of the pendulum; see again Fig. 6(a). The coordinates chosen to describe the system motion are the cart position $x_{c}$ and the absolute pendulum angle $\varphi $ resulting in the vector of generalized coordinates $\mathbf {q}=[x_{c},\varphi ]^{\mathsf {T}}$. Linear friction torque/force is considered for the revolute joint between cart and pendulum and also between ground and the cart, defined by the friction coefficients $d_{p}$ and $d_{c}$. The distance from the revolute joint to the pendulum’s center of gravity is abbreviated by $s_{p}$. The numeric values used for simulation are defined in the table in Fig. 6(b).

The goal of the optimization is to find the excitation force $F(t)$ that is best suited to generate measurements $\varphi (t)$ allowing the identification of $s_{p}$ and $d_{p}$. Again, the force $F(t)$ is constrained to the interval $[-F_{\mathrm{max}},F_{\mathrm{max}}]$. Incorporating a scrap function using $\varphi (t_{f}) = v_{c}(t_{f}) = \dot{\varphi }(t_{f}) = 0$ prevents from movements at $t>t_{f}$.

**Fig. 7:**
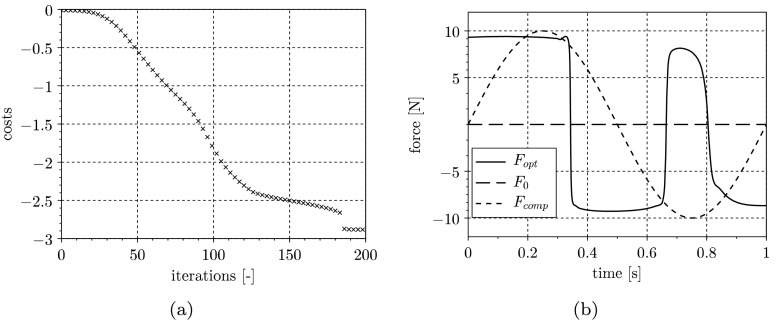
Convergence history for input optimization (**a**) and resulting excitation signal (**b**)

In Fig. 7(a), the convergence history for the input optimization is depicted. Figure 7(b) shows the resulting optimized input signal $F_{\mathrm{opt}}$, a signal $F_{\mathrm{comp}}$ used for comparison purposes, and the initial signal $F_{0}$. In Fig. 7(b), the signal $F_{\mathrm{comp}}$ is chosen as a sine wave with the period $T = t_{f}$, and the amplitude equals the force constraint $\vert F_{\mathrm{comp}}\vert = F_{\mathrm{max}} $. The plot of costs over iterations in Fig. 7(a) shows stable behavior and convergence at $n=200 \text{ iterations}$. At about $n=180 \text{ iterations,}$ we can observe a jump, which may result from the nonlinearity of the model structure.

**Fig. 8:**
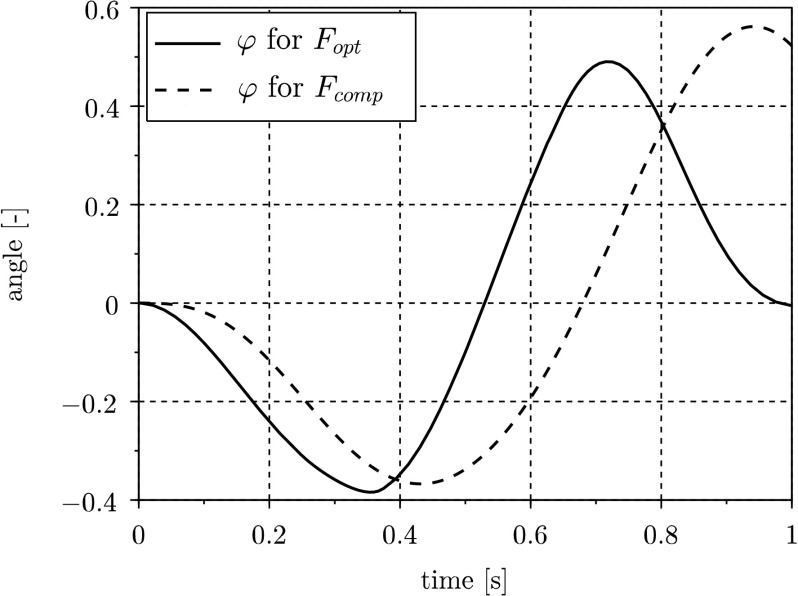
Pendulum angle $\varphi (t)$ for $F_{\mathrm{opt}}$ and $F_{\mathrm{comp}}$

**Fig. 9:**
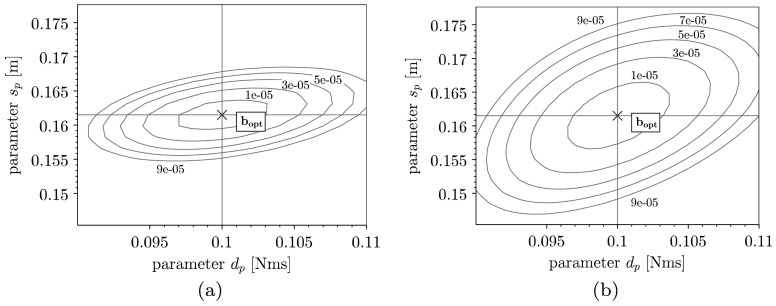
Contour plot of RMS error $E_{\text{RMS}}(\mathbf {b})$ for $F_{\mathrm{opt}}$ (**a**) and $F_{\mathrm{comp}}$ (**b**)

Unlike the previous example with only one parameter to identify, two parameters are now searched for. Comparing the error functions thus leads to three-dimensional plots or contour plots, where we can study differences in shapes for different excitation signals. Although the signals of the considered system output $\varphi $ (see Fig. 8) are comparable in amplitudes for $F_{\mathrm{opt}}$ and $F_{\mathrm{comp}}$, the RMS errors differ significantly. In Fig. 9, the contour plots for $F_{\mathrm{opt}}$ (a) and $F_{\mathrm{comp}}$ (b) are depicted, where both parameters $d_{p}$ and $s_{p}$ are varied in the range of $\pm 10\%$ of the nominal value. The contours represent the values of $E_{\text{RMS}}$ at levels that are chosen equally for both plots in Fig. 9(a) and Fig. 9(b). Analyzing the plots leads to three main differences. First, a small rotation of the functional can be detected, where the elliptical contours are more aligned with the coordinate axes in the case of using $F_{\mathrm{opt}}$. Further, a slight compression of the functional can be noticed, which leads to a worse condition of the optimization problem and therefore to poorer convergence for $F_{\mathrm{opt}}$. Finally, the main difference of both functionals is the curvature and hence the decreasing distance of level curves. The RMS error depicted in Fig. 9(b) therefore is flatter and, as explained in Fig. 5 of the previous example, more sensitive to biased signals of the measurements.

## Conclusion

The proposed method is mainly based on the assumption of optimality regarding the minimum standard deviation of identified parameters. It further allows us to set end conditions, which prescribe states at the end of an experiment. Looking at the example of the cart pendulum, this results in more robust measurement signals. Even when dealing with biased signals, more accurate parameter estimates are generated. Moreover, we assume that the proposed method can also handle different norms of the Fisher matrix ℳ in order to not only optimize the information content but also the condition of the optimization problem.
